# How the Immune System Deploys Creativity: Why We Can Learn From Astronauts and Cosmonauts

**DOI:** 10.3389/fpsyg.2021.582083

**Published:** 2021-04-26

**Authors:** Henderika de Vries, William Khoury-Hanold

**Affiliations:** ^1^Yale Center for Emotional Intelligence, Yale Child Study Center, Yale University, New Haven, CT, United States; ^2^Department of Immunobiology, Yale University School of Medicine, New Haven, CT, United States

**Keywords:** creativity, immune system, cosmonauts/astronauts, cognition, T cells, B cells, *Unheimlichkeit*, sports

## Abstract

In this interdisciplinary article, we investigate the relationship between creativity and the immune system; the creative features of the immune system and how the immune system and its role in regulating homeostasis might be related to creative cognition. We argue that within a multivariate approach of creativity, the immune system is a contributing factor. New directions for research are also discussed. When astronauts and cosmonauts venture into the new and extreme environment of outer space, their immune system needs to instantly adapt and find new answers to survive biologically and psychologically. Many astronauts report interest in creative activities and therefore represent an interesting group to investigate creativity in relation with the immune system. Little is known regarding (1) how the immune system interacts with and supports creative cognition and behavior, (2) if an individual’s immune system, interacting with cognition, adapts more originally to a new environment compared to another’s; in other words, if there is creativity in the domain of the immune system, and (3) the creative properties and functions of the immune system itself.

## Introduction

“An addiction to poetry is very generally the result of an uneasy mind in an uneasy body”

(Lord Byron, Letter, 4331, in Sandblom, 2009).

Given that the concepts of creativity and the immune system might not be familiar to those who study one but not the other, we will begin by defining these concepts. Next, we will argue that these seemingly lightyear distant concepts are actually closer than previously thought. We will discuss the connections between illness, the immune system and creativity and propose that the essence of illness is the experience of *Unheimlichkeit* which is the sense of “uncanniness” or “unhomelikeness” in one’s own body and in the world (Svenaeus, 2000). Finally, we will extend the framework of *Unheimlichkeit* to the extreme environment of space and discuss what we can learn about creativity from astronauts.

Creativity is the capacity to produce something new and original yet adapted to the constraints of a given situation (e.g., Sternberg and Kaufman, 2010; Amabile, 2018). Within a multivariate approach to creativity, the following factors are critical: knowledge; cognition, which includes intelligence, memory and attention; conative factors which includes motivation, tolerance for ambiguity, risk-taking, and emotions; personality; and context, which includes environment and cultural factors (e.g., cultural tightness vs. cultural looseness). Together, within a multivariate approach, these interacting variables contribute in varying degrees to the creative process (Sternberg and Lubart, 1995). That is because creativity is partly domain-general and partly domain-specific or even task-specific (Lubart and Guignard, 2004; Barbot et al., 2016) and depending on the given domain or task, the variables involved in creativity can vary and interact differently. For example, a creative scientist may require specific creative cognition processes more so than a creative dancer who might require more motoric ability or kinetic creativity. Creative artists score higher for the personality trait of neuroticism compared to those who are creative in their scientific work or in everyday creativity. Regardless, the key cognitive factors and personality traits that predict creative potential are “openness to experience” (McCrae, 1987; Dollinger et al., 2004) and “tolerance for ambiguity” (Stoycheva, 2010; de Vries, 2018) which are each essential for any domain or task (e.g., Batey and Furnham, 2006). Furthermore, de Vries found that people who are tolerant to ambiguity are also less judgmental. Taken together, a person who is tolerant for ambiguity remains curious rather than anxious in an uncertain or ambiguous situation thereby enhancing their creative performance (de Vries, 2018).

Each of the factors contributing to the creative process, such as personality traits and cognition, can manifest in and interact within a given environment. Ultimately though, when people engage in the creative process, they are activating a bodily and therefore biological process that is still poorly understood (Simonton, 2001). Therefore, our aim is to use extreme bodily conditions (e.g., those who experience disease or physical discomfort) and extreme environments (e.g., astronauts living and working aboard the International Space Station), to explore how the physical and related mental conditions of the body respond to, adapt, and recover from these extreme experiences; how these might be related to creative performance; and to use these examples to broaden our understanding of the concept of creativity (de Vries, 2018, 2019).

Existing research on the relationship between the body and creativity predominantly focuses on mental wellbeing (Jamison, 1993; Kaufman, 2014) and is confined to the neuroscience of creativity (Beaty et al., 2014, 2016; Jung and Vartanian, 2018). However, recent studies have expanded our understanding of the immune system beyond its role in controlling infections. In fact, we now know that the immune system is responsible for monitoring and maintaining the physiological equilibrium compatible with life known as “homeostasis” (Kotas and Medzhitov, 2015). It does so by communicating with every cell, tissue, and organ of the body to detect and address infection, injury, or stress (e.g., unfavorable nutrient concentrations, pH, or salt balance, etc.) (Kotas and Medzhitov, 2015). Therefore, the immune system is an essential component of the body’s ability to sense, respond to, adapt, and recover from internal and external perturbations. In line with this, dozens of parameters denoting immune system function change in response to the physical and psychological stress of spaceflight (Afshinnekoo et al., 2020).

Using an interdisciplinary approach, we address the relationship between the immune system and creativity by integrating insights from both of these fields (Repko and Szostak, 2020). Our aim is threefold: first, by examining individuals who experience “*Unheimlichkeit*” (e.g., those who suffer from chronic pain and astronauts), we will consider how the biology of the immune system should be included in the overarching theory of creativity; second, by integrating new insights from the field of immunology, we will discuss how the immune system serves as a principle regulator of both the body and the mind and propose new avenues of research for the field of creativity; and third, we will propose new perspectives, informed by creativity research, to broaden the field of immunology.

## Creativity and Bodily Experience: *Unheimlichkeit*

The field of psychology has long sought to understand processes related to internal and external factors that predict creative performance. Studying individuals and groups at the extremes of human experience exaggerates these principles which allows researchers to observe commonalities and patterns that might otherwise be too subtle to see in the broader population. While it is generally thought that very healthy individuals are creative (Flaherty, 2018), there are many examples of eminent artists who suffer from disease or chronic pain. According to one of the first authors on creativity and illness, Philip Sandblom, many artist’s lives and their creative works are heavily influenced by their bodily condition and the discomfort, pain, and debilitation it causes (2009). History abounds with many examples such as the world-famous Mexican painter Frida Kahlo (1907–1954) who suffered from chronic pain due to a spinal cord injury and polio; yet she kept painting even when her pain necessitated that she painted from her bed. Her body and pain are often depicted in her paintings and serve as creative external representations of her internal world. Another example comes from the French painter Pierre-Auguste Renoir (1841–1919) who wrote of his fellow French painter Henri Matisse (1869–1954)*:* “A lengthy martyrdom–his finger-joints were swollen and horribly distorted–yet he now painted his best works. While his body wasted away, his soul seemed to gain strength and he expressed himself with increasing ease” (Chatzidionysiou, 2019, p. 106). Finally, the Hungarian composer Bela Bartòk (1881–1945) suffered from polycythemia, which is a rare disease caused by a surplus of red blood cells leading to chronic pain and fatigue. Despite an enduring high fever, he wrote his famous swan song: Piano Concerto No. 3 in E major in the final months of his life.

Unfortunately, studies on the immune system and pain and creativity are few so it is difficult to answer the question how does the experience of pain relate to the creative process? A rare example of such a study found that expressive writing significantly diminished viral load in patients who were HIV^+^ (Petrie et al., 2004). An overview of art-therapy studies by Stuckey and Nobel (2010) shows that in general creativity is related to an improvement of vital signs whereas others maintain that more research is needed to determine if art therapy is an empirically supported treatment for illness (Holmqvist and Lundqvist Persson, 2012). This is currently poorly understood but warrants further research.

In her excellent work on motivation and the neurological underpinnings of creativity, Flaherty (2018) writes that suffering influences creative behavior by raising arousal and the motivation to be creative or, she suggests, by being distracted from the pain. The Swiss artist Paul Klee (1879–1940), who suffered from the autoimmune disease scleroderma, captured this notion well when he remarked: “I paint in order not to cry” (in Sandblom, p. 145). While the motivation to seek pleasure and avoid pain can be in competition, a smaller amount of pain can be endured for a larger reward (Flaherty, 2018). However, this does not explain how creative people can work enduring even extreme physical discomfort. The creative scientist and philosopher Arthur Schopenhauer (1788–1860) for example found the sensation of pain stronger and therefore more inspirational than pleasure.

We can observe an intimate connection between pain and pleasure anatomically. Neuroscience research on how pain and pleasure signaling are controlled in the brain show that these two sensations overlap in their neural circuitry and share a reliance on opioid and dopaminergic signaling (Leknes and Tracey, 2008). An explanation therefore could be that highly creative individuals, who are more tolerant for ambiguity, “judge” less pain and therefore can maintain their motivation. In other words, they undo the sensation of pain from its negative valence and are also open to the experience and sensation of pain. Another possible explanation is related to the fact that this neural circuit design allows for an individual to balance pleasure and pain depending on the reward. Pain can thus be switched off in favor of a pleasurable sensation such as gaining a reward if the benefit outweighs the cost. However, the reverse can also occur: a strong pain signal can override pleasure seeking behavior if the cost outweighs the reward (Leknes and Tracey, 2008). Artists who suffer from chronic pain might be exceptionally capable of maintaining strong motivation to seek the pleasure of their reward (creating) so much so that it over-rides the pain signals that might otherwise inhibit their creative process.

While this framework helps to explain the neurological relationship between chronic pain and creativity, it does not allow us to easily incorporate other bodily experiences also known to be associated with creativity. For example, the world-renowned cosmologist and physicist Dr. Steven Hawking (1942–2018) was remarkably creative in the domain of science. He also lived and worked for more than 50 years with amyotrophic lateral sclerosis (a motor neuron disease), which first appeared in his twenties. Despite this, he made several paradigm-shifting contributions to the field, defining the Hawking radiation released by black holes and unifying the general theory of relativity with quantum mechanics.

Historically, researchers studied creative people such as artists and scientists, using the framework of health and disease. However, we believe this binary takes an overly simplistic, problematic and ableist view of “health.” Furthermore, it fails to define the commonalities shared between seemingly different creative people. To find these commonalities, we must first generalize the experience of illness. To do so, we now turn to the philosopher Fredrik Svenaeus who defines the essence of illness as *Unheimlichkeit* which is the sense of “uncanniness” or “unhomelikeness” in one’s own body and in the world (Svenaeus, 2000). One experiences *Unheimlichkeit* when one loses their ability to understand their own embodiment and the ability to experience and make meaning of the things and people around them (Svenaeus, 2000). Illness, regardless of its etiology and manifestation, dislodges a person’s sense of their own body as familiar and coverts it into something that is quite opposite to homelike: dangerous, strange, incomprehensible and alien (Svenaeus, 2000). Illness activates this sense of alienation or otherness to levels that are “obtrusive and merciless” giving rise to feelings of helplessness (Svenaeus, 2000). Using this framework, we now see how artists suffering from chronic pain; Dr. Hawking slowly losing his motor abilities; athletes training their bodies to perform unnatural and unintuitive motions and gestures; all experience *Unheimlichkeit*.

This suggests that creativity is linked to both the experience of *Unheimlichkeit* and the subsequent mental and physical adaptations one makes to restore their sense of “homelikeness” and their ability to understand their world. While pain or disability can activate *Unheimlichkeit*, there are many ways one might feel unhomelike in their own body; for example, when experiencing the new and extreme environment of space. We are at a unique moment in our 200,000 years history as humans: we are traveling and living in space for the first time with plans to establish new settlements on the moon and Mars in the coming decades. Space is a unique and completely novel environment beyond the confines of our home planet that demands of astronauts and cosmonauts the ability to generate solutions to new problems. The space stations that astronauts live and work in are themselves hubs of research and discovery. Finally, astronauts and cosmonauts have described the “Overview effect” (White, 2019), which is a transformative experience that occurs when one looks out of their spacecraft’s windows and sees our tiny and fragile home planet suspended against the backdrop of the rest of the universe. Therefore, during space travel, astronauts and cosmonauts experience both a literal “unhomelikeness” and the philosophical *Unheimlichkeit* upon leaving the gravitational and metaphorical grounding of Earth. Astronaut Meir (2020) recalls that she felt a strange experience when her brain seemed to “flip-flop” upside down when it finally adjusted to its new orientation in space.

Therefore, astronauts experience a unique form of *Unheimlichkeit* that might underlie their creative performance. Interestingly, astronauts report an increase in creative interests during and after travel to outer space. In fact, most of the 500+ astronauts who ventured into space are also remarkably creative in various other domains including painting (e.g., Stott, 2019), music (e.g., Stott, 2019), and photography (e.g., Stott, 2019). Some even became professional artists after their missions such as Alexei Leonov (1934–2019) who was the first human to not only walk in outer space but also to create art in outer space, sketching the sunrise using color pencils. He was also known to make charcoal portraits of his crewmates aboard their Voskhod 2 spacecraft. His missions inspired him in his well-known work afterward. It is currently unknown if the intense experience of space travel and experiencing the “Overview effect” (White, 2019) changes an astronaut’s creative potential and this deserves further research. Tracking changes in creative performance before, during and after space flight (and i.e., the experience of *Unheimlichkeit)* will be a powerful tool in further defining the cognitive and physiological components of creativity.

Here we see how openness and acceptance to the deeper experience and sensation of *Unheimlichkeit* seems to provide a catalyst to the creative process. However, there are still many empirical unknowns concerning the dynamics and the physiological adaptations to the uncanny. To understand how the bodies of eminent creative individuals respond to, adapt, and recover from *Unheimlichkeit* or other extreme experiences, we now turn to the immune system as the body’s central regulator of homeostasis.

## The Role of the Immune System in Controlling Homeostasis, the Creative Brain and Behavior

By identifying creative individuals who experience *Unheimlichkeit*, we can also study what changes occur in the body during and after these experiences to learn more about the biological underpinnings of the creative process. While the brain and cognition have been the rightful focuses of past research, we argue that the immune system should feature more prominently in future creativity research given its emerging role in modulating behavior and cognition. In response to infection, immune cells become activated and release inflammatory factors, known as cytokines, that allow them to further activate and coordinate appropriate immune responses to clear the infection and repair damage. However, these cytokines also act on the brain to induce a set of “sickness behaviors.” These include loss of appetite, loss of libido, altered sleep, social withdrawal, fatigue, and altered cognition and mood (Dantzer and Kelley, 2007). The coordination between the immune system and the brain enhances the likelihood of survival because resources that would otherwise support nonessential programs, such as growth and reproduction, are instead conserved and allocated toward resource costly programs that support immune defenses against pathogens (Wang A. et al., 2019).

While the immune system has traditionally been studied in the context of infection, it is now clear that it plays a broader and vital role in regulating homeostasis throughout the entire body. In fact, the proper function of every organ and tissue such as muscle (Tidball, 2017), bone (Takayanagi, 2007), liver (Kubes and Jenne, 2018) and many others depends on immune cells that reside in the tissue and monitor and correct perturbations to homeostasis (Kotas and Medzhitov, 2015). Deviations from homeostasis, such as the perturbations experienced during infection, injury, stress and even space travel are both sensed by and rectified by the coordinated efforts of the immune system. Even non-immune cells of the body respond to inflammatory perturbations by producing factors that allow them to coordinate–under the direction of the immune system–to resolve the problem and return to homeostasis (Krausgruber et al., 2020).

We argue that astronauts provide a unique opportunity to address these questions given that space travel is a discrete event which causes extreme perturbations to homeostasis and where the immune system represents the first “protective shell” of the space traveler’s environment (Whiteley and Bogatyreva, 2008). Given that the timing of *Unheimlichkeit* and creative inspiration is known, researchers can track changes to the immune system before, during and after space flight and study how that relates to changes in creativity. Crews on space missions experience many psychological changes due to the biotic and abiotic stresses of space travel such as microgravity, radiation, altered nutrition, confinement, a busy work schedule, disrupted circadian rhythm, and the flight itself (Crucian et al., 2013; Thiel et al., 2017). As a result, astronauts experience cardiovascular dysregulation, bone demineralization, muscle atrophy, altered neuro-vestibular perception leading to extreme nausea, increased cancer risk, liver disease, nervous system and cognitive impairments, and immune system dysfunction (Afshinnekoo et al., 2020). Strikingly, half of the astronauts during the early Apollo spaceflights in the 1960s and 1970s developed bacterial and viral infections during and after spaceflight. More detailed studies have revealed that space travel is associated with broad changes throughout the immune system. During space flight, the immune system enters a period of broad dysregulation that includes reductions in the numbers and functionality of natural killer cells and T cells but increases in the numbers and functionality of neutrophils and monocytes (Crucian et al., 2013, 2015). Compared to pre-flight levels, parameters of the immune system adapt to the new environment and establish a new set point that persists during long-term space flight (Crucian et al., 2015; Thiel et al., 2017) and even for some time after returning to Earth (Buchheim et al., 2019). These changes have dramatic consequences on an astronaut’s ability to respond to infection so much so that reactivation of latent herpesvirus infections remains a frequent problem (Crucian et al., 2020).

In addition to anti-microbial immunity, the immune system also controls how the brain adapts to space flight. Using a mouse model, researchers showed that low-dose radiation similar to the levels encountered in deep space results in deficits in learning and memory formation (e.g., novel object recognition and fear-extinction response), which ultimately led to distress behaviors (e.g., social avoidance and behaviors resembling post-traumatic stress syndrome) (Acharya et al., 2019). Remarkably, many of these detrimental effects could be prevented by blocking the activity of a population of brain-resident immune cells known as microglial cells (Krukowski et al., 2018). Another brain-resident immune cell known as T cells also play an important role in regulating learning and memory (Kipnis et al., 2012) and social behaviors (Filiano et al., 2016; Reed et al., 2020). In mouse models, the amount of time a subject spends with either a novel inanimate object or a novel mouse is quantified and used to approximate sociability. Whereas normal mice prefer to interact with each other, subjects with defects in meningeal T cells exhibited anti-social behaviors by spending more time interacting with the inanimate object (Filiano et al., 2016; Reed et al., 2020). To assess learning and memory in mice, subjects are allowed to explore one half of a simple Y-shaped maze. When introduced to the other half of the maze, normal mice were more likely to explore the novel arm compared to the familiar arm. However, mice lacking meningeal T cells had short-term memory defects and were less likely to explore the novel arm of the Y-maze (Ribeiro et al., 2019). Taken together, these findings further suggests that T cells are important in generating the “openness to experience” that is so vital to the creative process. As of yet, these questions have only been investigated in mouse models of cognition and behavior; therefore, further research involving human subjects will be vital in further pursuing the role of T cells in creative cognition.

While space travel is known to alter the number of functions of T cells circulating in the blood (Crucian et al., 2013), it is unknown whether brain-resident T cells, such as those that modulate learning, memory, social behavior and openness to new experience, are also affected by space travel. In the future, as mouse and human research on board spacecrafts become more sophisticated, there will be a remarkable opportunity to study how the immune system of the brain changes before, during and after space travel and how this affects the cognitive and behavioral contributors to creative performance such as openness to experience, mind-wandering and spontaneous thinking (Beaty et al., 2016), short/long-term memory, concentration and flow. In the meantime, terrestrial-based space analogs, such as the Mars500 mission, overwintering in Antarctica, and the Hawaii Space Exploration Analog and Simulation (HI-SEAS) Habitat (Mahnert et al., 2021) offer researchers the opportunity to not only approximate the stresses of space travel but also isolate variables that contribute to the physiological, cognitive and behavioral changes associated with space travel (Pagel and Choukèr, 2016).

## The Immune System and the Creative Process

Having discussed how insights from how the immune system controls homeostasis and cognition and how these might enhance our understanding of creativity, we now consider how theories of creativity might apply to immunology and inspire new research perspectives. However, it is important to make the distinction between what are fundamentally biological processes and human creative cognition. Previously, Campbell’s Blind Variation and Selective Retention (BVSR) model of creativity used Darwin’s theory of evolution through natural selection as a representation for creative thought (Campbell, 1960). However, the “Blind Variation” component was often rejected (Simonton, 1999, 2011) because it failed to accommodate human volition; therefore, a “Sighted Variation” model was proposed instead (Sternberg, 1998). Current literature in physics questions whether the idea of blind variation actually exists at all given that there are patterns of entanglement and relationships even at the most basic level of atoms, as explained in the “rule space relativity” (Wolfram, 2020).

In line with the above, we maintain that some biological processes demonstrate emergent creative properties. There are different ways the creative process is described. The most simple one is a process of divergence, and convergence (e.g., Klahr and Dunbar, 1988). Divergent thinking represents an exploratory phase when the generation of many ideas in different directions and fluency (i.e., the amount of ideas) are key. In the convergent thinking phase, ideas are integrated and converged into one or possibly more “right” (i.e., adapted to a problem) answers. Other steps of the creative process include for example: (1) finding and formulating a problem, (2) acquiring knowledge, (3) gathering potentially related information, (4) taking time for incubation, (5) generating a large variety of ideas, (6) combining these ideas in unexpected ways, (7) selecting the best ideas, and (8) externalizing an idea (Sawyer, 2011). Studies by Botella et al. (2013; 2018; 2019) show that in reality the creative process is not sequential nor linear and depends on the domain of creativity as well. These researchers determined from diaries of artists the dynamic nature of the creative stages such as immersion, search, thinking, trials, inspiration, insight, ideation, combination, abandonment, selection, technique, precision, realization, judgment, finalization, break, and completion. Here, we will describe how adaptive immune cells, specifically B cells, relate the key features of their development to the creative process.

**FIGURE 1:**
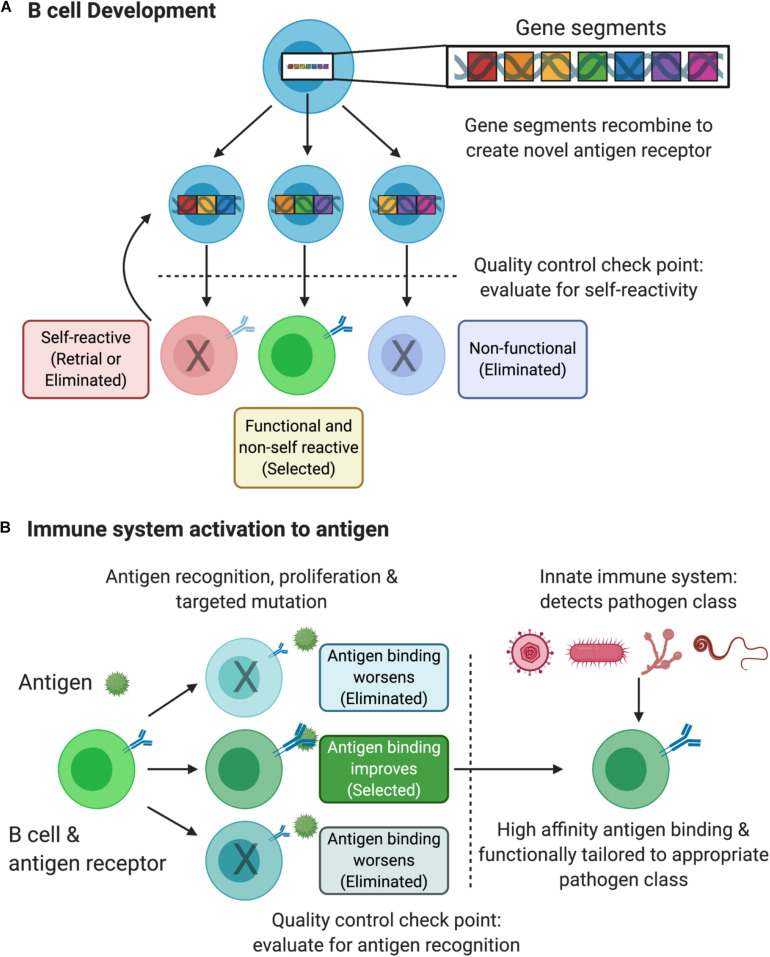
B cell receptor development and refinement. **(A)** Developing B cells in the bone marrow randomly recombine DNA gene segments to encode a novel and unique receptor specific to a given antigen. At the first quality control check point, B cells that fail to generate a functional receptor are eliminated from the repertoire. B cells that generate a self-reactive antigen receptor undergo a period of “receptor editing” to attempt to lose this self-specificity. B cells that generate a functional receptor that does not recognize self antigens are selected, complete their development and egress from the bone marrow. While distinct, T cell development in the thymus shares these fundamental and analogous features. **(B)** Following immune system activation and antigen encounter, B cells that recognize the antigen proliferate and undergo further antigen receptor refinement. Random mutations are introduced to the DNA encoding the antigen receptor thereby changing its ability to recognize the antigen. The second quality control check point assess the B cell receptor’s binding strength to the antigen. Those B cells with beneficial mutations are selected while those with deleterious mutations are eliminated from the repertoire. Finally, information gathered by the innate immune system induces qualitative changes to the B cell antigen receptor to better suit the response to a given class of pathogen infection (e.g., viral vs. bacterial vs. fungal vs. helminth). Overall, this process improves the precision of antigen recognition and tailors the B cell response to more efficiently clear the infection. Figure created with BioRender.com.

A fundamental biological problem that multicellular organisms must solve is how to distinguish their own “self” cells and molecules from those of other “non-self”–often infectious–organisms (*formulating a problem*). The immune systems of all animals and plants make this distinction by broadly defining non-self, microbial organisms by their unique molecular features that are distinct and absent from their own tissues. For example, components of bacterial cell walls or elements of viral genomes are distinctive to those organisms and generally not found in animal or plant tissues outside the context of infection. The molecular receptors animals and plants use to broadly detect infectious agents are encoded in their germline DNA meaning that they are an inherited and “innate” component of the immune system whose refinement occurs on an evolutionary timescale (Kawai and Akira, 2010). These innate immune receptors also allow the immune system to gather broad information by classifying the infection as either bacterial, fungal, viral or helminth (*gathering information*). This allows the immune system to tailor its response to a given class of pathogen and more effectively clear the infection. In addition to the innate immune system, vertebrate animal immune systems evolved an even more discerning set of tools capable of recognizing nearly any biological compound in the world with exquisite specificity that goes well beyond the broad discrimination described above (Flajnik and Kasahara, 2010). Specialized cells known as T and B cells use a creative process to construct unique molecular receptors to identify non-self molecules, referred to as “antigens.” For simplicity’s sake, we will focus on B cell development (Figure 1A) but T cells undergo an analogous process that shares many similar fundamental features. Each B cell, during its development, constructs a unique receptor, known as an antibody, *de novo* from pieces of germline encoded DNA called “gene segments.” These gene segments are not functional individually but rather are the basic building materials B cells use to construct a new antigen receptor. During a process known as somatic recombination, gene segments are cut and spliced together to form a functional stretch of gene-encoding DNA (*idea generation*). In the process, the junctions between the newly spliced gene segments are mutated such that no two cells are likely to produce identical receptors even if they happen to choose the same gene segments to recombine (*combining ideas in unexpected ways*). Finally, each “half” of a B cell receptor is assembled independently and must be paired with another set of randomly assembled gene segments. Therefore, using random recombination of a limited number of gene segments and random mutations, B cells can produce an estimated 10^13^ to 10^18^ unique antigen receptors! While individual B cells construct one unique antigen receptor and can therefore only recognize one antigen, taken as a population–approximately 10 billion B cells in the human body–their combined recognition capacity yields a staggering repertoire with immense sensory capacity (Murphy and Weaver, 2017).

Newly constructed antigen receptors are then screened by a series of quality control checkpoints that evaluate them for basic functionality and proper specificity (*selecting the best ideas*). Given that gene segments are combined in a random manner, the B cells risk generating receptors that might recognize the body’s own biological compounds (i.e., “self antigens”). Doing so would result in auto-immune disease where the immune system recognizes and destroys its own tissues (e.g., multiple sclerosis or rheumatoid arthritis). Developing B cells that generate these self-reactive receptors are either eliminated from the repertoire in order to prevent autoimmunity or the self-reactive B cell undergoes a period of “receptor editing” (*precision*). During this process, gene segments are again randomly recombined but from a pool that is reduced to exclude those gene segments that previously failed the first quality control check point. Interestingly, this selection process mirrors cultural aspects of creativity such as “cultural tightness – looseness.” If the selection criteria are too tight, then the repertoire of antigen receptors might be too limited and fail to recognize pathogens when they are present; and if the criteria are too loose, then the repertoire would be too broad and therefore erroneously recognize self-antigens as non-self, leading to autoimmune disease. Finally, in a manner similar to the creative process, this molecular process involves risk given that segments of DNA are cut and rearranged with the intervening stretches removed altogether. This permanently changes the genetic landscape of the developing cell and, if not tightly controlled, can lead to cancer (Shaffer et al., 2002). In the event of a successful rearrangement, the B cell completes its maturation (*finalization*) and exists the bone marrow having generated a novel antigen receptor that will detect a non-self antigen of a given specificity (*externalizing an idea*) (Figure 1A).

In the event that the immune system is activated in the presence of a non-self antigen, such as during an infection, the B cell that recognizes that antigen will proliferate thereby amplifying the use of the successfully developed antigen receptor. Remarkably, further refinement of this B cell receptor takes place at this stage (Figure 1B). Responding B cells introduce additional random mutations to the gene encoding the B cell receptor, which will alter the ability of the receptor to bind to and detect the antigen. An additional quality control check point assesses these modified receptors (*judgment*) and selects those that recognize and bind with even greater strength and eliminate those that weakly bind to the antigen (*again selecting the best ideas*).

In summary, the immune system employs many features of creativity to collectively solve the following problem: how can a biological system distinguish self antigens from the myriad of non-self antigens that exist in the biological universe given limitations on the size and complexity of its genome? It does so by randomly combining simple building blocks to produce a unique tool (*divergent production and exploration of various directions*) that is subsequently evaluated for its functionality and for its ability to bind non-self antigens (*convergence toward one or several solutions*). This mitigates the risk involved in an otherwise random and potentially dangerous process. Following exposure to antigen and innate immune identification of pathogen class (*gathering broad information*), B cell receptors are further refined to improve the quantitative binding properties to the antigen and the qualitative features that are most suited to clearing a given class of pathogen.

Astronauts and cosmonauts, whose immune systems must creatively adapt in the extreme environment of space, provide a novel and important population in which to further study the creative features of the immune system. Intriguingly, space travel was shown to adversely affect T cell development in the thymus of astronauts (Benjamin et al., 2016). Complementary studies involving mice aboard the International Space Station identified that the stress of microgravity led to defects in T cell development and generation in the thymus (Horie et al., 2019). How space travel ultimately affects the creative process by which T cells generate their antigen recognition repertoire–the combined capacity of all individual T cell clones have in recognizing antigens–remains incompletely understood. Animal models of extreme gravitational stress suggest that space travel might alter antigen receptor generation and quality control in T cells (Ghislin et al., 2015; Fonte et al., 2019), but corresponding studies in astronauts are rare. One such study showed that the populations of T cells that recognize common herpesviruses are unaffected by space travel (Crucian et al., 2015) but other T cell populations of differing recognition capacities remain to be tested. Furthermore, whether the previously discussed brain-resident T cells that control learning, memory and openness to new experience are affected by space travel is an intriguing but open question.

While B cell numbers are stable before, during and after spaceflight (Spielmann et al., 2019), emerging evidence strongly suggests that their antigen recognition repertoire does change during this time. Studying a small group of astronauts aboard the International Space Station over a period of several months, Buchheim et al. (2020) found that space travel affects B cell development (Figure 1A). Specifically, the frequencies of various spliced gene segment combinations and the mutations at the junctions between spliced gene segments were significantly altered during the flight in two of the five astronauts studied (Buchheim et al., 2020). These same parameters were remarkably stable in the other three astronauts and ground-based control subjects. As with individuality in creativity, this suggests that individual adaptations to space extend to somatic recombination and B cell development. Unexpectedly, this study also showed that even before space flight, features of the astronaut’s B cell antigen recognition repertoire were already significantly different from the ground-based control subjects and that these differences were not likely due to differences in antigen exposure (Buchheim et al., 2020). Going forward, it will be exciting to determine (1) whether the selection and training of astronauts biases the B cell repertoire or vice versa; (2) whether changes in the creative immune system correspond to changes not only in immunity but also in the creative performance of astronauts; and (3) whether similar changes in the B cell repertoire occur in other scenarios involving *Unheimlichkeit* and dramatic changes in creative performance or whether they are unique to astronauts and cosmonauts living in space.

## Discussion

In this article, we argued that the immune system is central to and is a catalyst for creativity. Specifically, based on the literature on illness and creativity, and the immune system’s role in maintaining homeostasis, we suggest that the immune system is a component of creativity and one of its underlying biological mechanisms. Using the concept of *Unheimlichkeit*, we argued that the immune system, through its role in maintaining homeostasis, responds and adapts to new environments and that this is a critical feature of creative people. We also discussed how the immune system plays a critical role in maintaining homeostasis in the brain and has been shown to modify cognition, learning, memory and behavior (Dantzer and Kelley, 2007; Ribeiro et al., 2019). It is therefore possible that the immune system also plays a role in creative cognition and behavior and affects the personality trait “openness to experience” (Ribeiro et al., 2019). Next, we argued that there are creative properties of the immune system itself. We found that the immune system mirrors (surprisingly) many creative features and processes. Finally, using astronauts and cosmonauts as a unique study population, we propose future research that might determine whether some individual’s immune system adapts more originally or creatively to a new environment compared to others. For example, do more creative individuals develop more diverse and original adaptations (or illnesses)? In other words, are some individuals more creative in the domain of the immune system than others?

**FIGURE 2:**
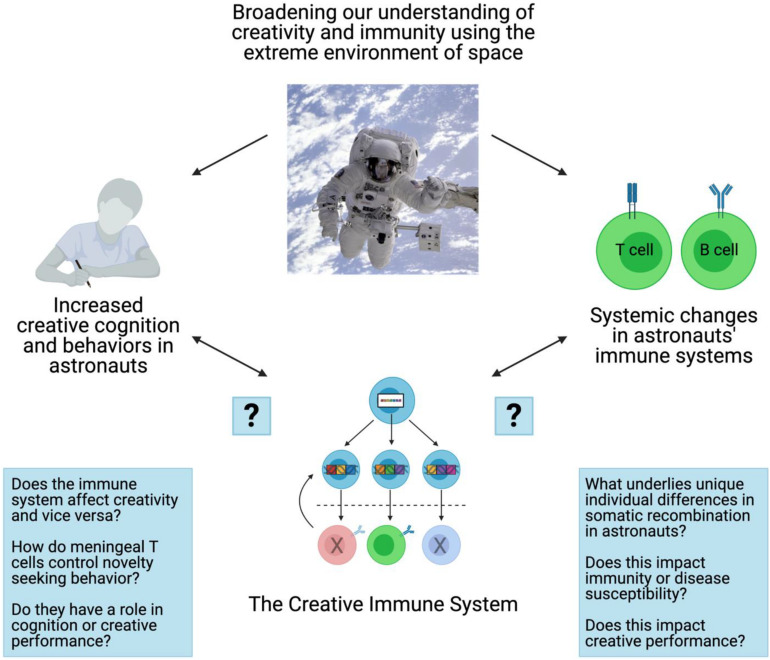
Summary schematic and outstanding questions. Space travel leads to profound changes in creative behaviors and various parameters of the immune system. The immune system itself, namely T and B cells of the adaptive immune system, exhibit features of the creative process which is also altered by space travel. Whether and how these phenomena are connected is unknown; specific outstanding questions are also posed in this summary schematic. Figure created with BioRender.com.

As previously mentioned, investigating the immune responses, creative performance, and mental/physical wellbeing of astronauts before, during and after space flights could provide valuable insights into how these physiological changes relate to an astronaut’s level and process of creativity. In line with this, recent data show that when astronauts’ sense of “homelikeness” aboard the International Space Station was enhanced through improvements in diet, stress management, mental health, exercise and physical health, their immune dysregulation was ameliorated (Crucian et al., 2020). Future studies might leverage these kinds of interventions to more precisely determine which biotic and abiotic stressors control physiological adaptations to space travel. A practical difficulty in conducting research with astronauts and cosmonauts are the relatively small sample sizes of participants and the logistical constrains space travel imposes on research. Therefore, future research might start with case studies of individual astronauts and track changes in their immune system compared to other variables like personality, values and creative cognition in order to define new directions. Coupled with terrestrial-based space analogs (Pagel and Choukèr, 2016), future work might also determine the relative roles variables such microgravity, social isolation, circadian disruption, the “Overview effect,” etc., have on, for example, B cell antigen receptor development. Finally, these kinds of studies might answer the question of the direction of the relationship between the immune system, wellbeing and creativity (Figure 2).

Additional outstanding questions are whether changes in creative performance or expression is associated with changes in immune function at steady-state (e.g., immune system controlling the homeostatic regulation of digestion, absorption, and distribution of nutrients) (Kotas and Medzhitov, 2015) or during infection or injury (e.g., overt immune activation to bacterial infection). Given the role of the immune system in controlling sickness behaviors (Dantzer and Kelley, 2007) it is tempting to speculate that immune responses to infection and injury would affect creativity in an analogous manner to ways that other forms of stress, or other situations that evoke a profound sense of *Unheimlichkeit*, affect creativity (Wang X. et al., 2019).

Overall, we suggest that the immune system’s role in regulating homeostasis is a contributing factor within the multivariate approach to creativity (Sternberg and Lubart, 1995). This means that there might be a particular domain of creativity in which this variable is especially required. Such a domain could be the extreme performance observed in sports. For example, a sprinter breaking the record in the 100 meter event also produces a unique and adapted performance to an imagined extreme environment. On a racing track, there is no real danger in the environment (e.g., a predator) that someone needs to run from. While preparing for a unique, novel and adapted performance, it is known that the immune systems of top athletes are profoundly affected by the physical strain of their intense training regimens and that this adversely affects their ability to respond to infections (Gleeson and Pyne, 2016). Furthermore, T cells play a critical role in the muscle repair response following injury (Burzyn et al., 2013). In this sense if athletes might express creativity by accomplishing a unique and adapted top performance, then the variable of the immune system would be a major contributing factor. This represents an addition to the new emerging domain of creativity and sport (Vaughan et al., 2019; Richard and Runco, 2020).

Concerning immunology research, the perspective that complex cellular systems have emergent properties that mirror creative processes broadens our understanding of systems biology and might therefore inspire new research directions. For example, do the immune systems of highly creative people function differently from individuals with lower creative potential? Ongoing research programs should seek to better understand the role of the immune system and cognition, especially the cognitive processes that are involved in the generation of creativity. For example, given the role of T cells in exploration and memory (Ribeiro et al., 2019), how does the lymphocyte repertoire of antigen recognition receptors affect creative potential and are there differences in these repertoires between highly creative and non-creative individuals?

Finally, given the increasing examples of a post COVID-19 syndrome that mimics aspects of other chronic conditions such as myalgic encephalomyelitis/chronic fatigue syndrome (ME/CFS) (Stam et al., 2020), it will be important to document how these peoples’ immune systems change, how their experience of *Unheimlichkeit* evolves, and how it relates to their creative performance. This knowledge could be applied to secure the wellbeing of astronauts and cosmonauts who also experience debilitating mental and physical fatigue (Scheuring et al., 2015).

Dr. Homburger predicted in the preface of Sandblom’s book in 1982 that 1 day an investigator will clear up the mystery of how the soma and psyche interact. An integrated understanding of the immune system and creativity might very well be a start in this direction.

## Author Contributions

Both authors listed have made a substantial, direct and intellectual contribution to the work, and approved it for publication.

## Conflict of Interest

The authors declare that the research was conducted in the absence of any commercial or financial relationships that could be construed as a potential conflict of interest.
